# Supplementary material for: Data-driven prediction of continuous renal replacement therapy survival
Source: Nat Commun. 2024 Jun 27;15:5440. doi: 10.1038/s41467-024-49763-3 (PMC11211317; doi:10.1038/s41467-024-49763-3)
Supplement: Supplementary file 1 — Supplementary Information [file 41467_2024_49763_MOESM1_ESM.pdf]

Data-driven prediction of continuous renal replacement therapy survival

Davina Zamanzadeh[1], Jeffrey Feng[2], Panayiotis Petousis[3], Arvind Vepa[2], Majid Sarrafzadeh[1], S Ananth Karumanchi[4], Alex A. T. Bui[2*†], Ira Kurtz[5†]

[1]Department of Computer Science, University of California, Los Angeles, Log Angeles, 90095, California, United States.
[2]Medical & Imaging Informatics Group, University of California, Los Angeles, Los Angeles, 90095, California, United States.
[3]Clinical and Translation Science Institute, University of California, Los Angeles, Los Angeles, 90095, California, United States.
[4]Department of Medicine, Cedars-Sinai Medical Center, Los Angeles, 90048, California United States.
[5]Department of Medicine, David Geffen School of Medicine, University of California, Los Angeles, Los Angeles, 90095-1689, California, United States.

*Corresponding author(s). E-mail(s): buia@mii.ucla.edu;
†These authors jointly supervised this work.

Supplementary information

Supplementary Algorithm 1 Train, tune, and evaluate a model to predict if a patient should be placed on CRRT.

$\mathbb{D}_{\text{train+val}} \Leftarrow$ choose from {UCLA: CRRT, UCLA: Control, Cedars: CRRT }
$\mathbb{D}_{\text{test}} \Leftarrow$ choose from {UCLA: CRRT, UCLA: Control, Cedars: CRRT }
if $\mathbb{D}_{\text{train+val}} = \mathbb{D}_{\text{test}}$ **then**
    $\mathbb{D}_{\text{train}}, \mathbb{D}_{\text{val}}, \mathbb{D}_{\text{test}} \Leftarrow \mathbb{D}_{\text{train+val}} * [0.6, 0.2, 0.2]$ ▷ Split dataset into 60/20/20% respectively.
else
    $\mathbb{D}_{\text{train}}, \mathbb{D}_{\text{val}} \Leftarrow \mathbb{D}_{\text{train+val}} * [0.8, 0.2]$ ▷ Split dataset into 80/20% respectively.
end if
$w \Leftarrow \{1,2,3,4,5,6,7,10,14\}$ days ▷ look-back window size
$m \Leftarrow$ {lgbm, xgb, rf} ▷ model type
$i \Leftarrow$ {mean/mode, k nearest neighbors} ▷ imputation method
$f \Leftarrow$ {k best, correlation threshold} ▷ feature selection method
$\mathbb{H} \Leftarrow \{w, m, i, f\}$ ▷ Set hyperparameter grid
function TUNING($\mathbb{H}$, $\mathbb{D}_{\text{train}}$, $\mathbb{D}_{\text{val}}$)
    metrics $\Leftarrow \{\}$
    for $w_j, m_j, i_j, f_j \in \mathbb{H}$ **do**
        Train $m_j(f_j(i_j(\mathbb{D}_{\text{train}})))$
        metrics $\overset{+}{\Leftarrow}$ Evaluate $m_j(f_j(i_j(\mathbb{D}_{\text{val}})))$
    end for
    $m^* \Leftarrow m_j$ such that corresponding metric is max/min (best)
    return m^*
end function
$m^* \Leftarrow$ TUNING($\mathbb{H}$, $\mathbb{D}_{\text{train}}$, $\mathbb{D}_{\text{val}}$)
metrics $\Leftarrow$ Evaluate $m^*(\mathbb{D}_{\text{test}})$
subpopulations $\Leftarrow$ (heart, liver, infection) $\times$ (male, female, race)
for subpopulation in subpopulations **do**
    metrics by subpopulation $\Leftarrow$ Evaluate $m^*(\mathbb{D}_{\text{test}}$*subpopulation)
end for

Supplementary Table 1 Optimal parameters (model, window before CRRT initiation, and feature selection correlation threshold) and number of features before and after training for all experiments. Feature selection with a correlation threshold was optimal for all experiments. Note that for each experiment, the feature counts correspond to the features that were available at the optimal window after hyperparameter tuning. The feature counts after training also correspond to the optimal feature selection method after hyperparameter tuning.

				Number of features before training		Number of features after training	
Experiment	Model	Window (days)	Correlation threshold	Raw features	Total features (engineered)	Raw features	Total features (engineered)
UCLA Model	lgb	4	0.065	2302	8529	212	235
Cedars Sinai Model	rf	14	0.045	1287	4103	176	239
UCLA + Cedars Sinai Model	lgb	7	0.045	2791	10892	173	254
UCLA + Cedars Sinai + Control Model	xgb	14	0.015	3220	12966	810	1143
UCLA Model, Feature Intersection of All Cohorts	xgb	5	0.055	503	794	190	220
UCLA Model, Feature Intersection of UCLA and Cedars Sinai	xgb	4	0.04	623	906	279	353
Cedars Sinai Model, Feature Intersection of All Cohorts	rf	14	0.035	556	861	231	321
Cedars Sinai, Feature Intersection of UCLA and Cedars Sinai	rf	14	0.015	662	971	475	685
UCLA + Cedars Sinai Model, Feature Intersection of All Cohorts	lgb	6	0.01	514	810	401	621
UCLA + Cedars Sinai Model, Feature Intersection of UCLA and Cedars Sinai	lgb	7	0.035	648	952	207	248
UCLA + Cedars Sinai + Control Model, Feature Intersection of All Cohorts	xgb	14	0.03	556	861	270	377

Supplementary Table 2 Performance measured by ROCAUC, PRAUC, and Brier Score when applying models to subgroups of respective test sets. Subgroups were categorized by three types of ICUs, which captured the vast majority of CRRT patients: medical ICU, cardiac ICU, and surgical ICU. The types of ICU were obtained from the name of the department that delivered CRRT, which was provided for all patients within the UCLA: CRRT and Cedars: CRRT cohorts. Only patients who were treated in an ICU that was explicitly one of the three types were considered for analysis. Mixed ICUs were not considered. Other specialized units such as neuro-ICU were also not considered due to limitations in sample size. All test sets included patients who were on CRRT for up to seven days.

Model	Evaluation Dataset	Subgroup	Count (% positive)	AUROC (95% CI)	PRAUC (95% CI)	Brier Score (95% CI)
UCLA Model	Holdout Test	All	425 (52.2%)	0.84 (0.80-0.87)	0.86 (0.82-0.90)	0.17 (0.15-0.18)
		Cardiac ICU	35 (48.6%)	0.91 (0.79-1.00)	0.93 (0.83-1.00)	0.13 (0.08-0.18)
		Medical ICU	124 (46.0%)	0.85 (0.78-0.91)	0.81 (0.69-0.91)	0.16 (0.13-0.20)
		Surgical ICU	212 (61.3%)	0.82 (0.76-0.87)	0.89 (0.85-0.93)	0.17 (0.14-0.20)
	External Validation	All	1788 (51.6%)	0.63 (0.61-0.66)	0.64 (0.60-0.67)	0.24 (0.24-0.25)
		Cardiac ICU	515 (50.1%)	0.61 (0.56-0.65)	0.63 (0.56-0.69)	0.24 (0.23-0.25)
		Medical ICU	531 (41.2%)	0.59 (0.54-0.65)	0.51 (0.45-0.58)	0.24 (0.22-0.25)
		Surgical ICU	509 (66.6%)	0.66 (0.61-0.71)	0.77 (0.73-0.83)	0.25 (0.24-0.26)
Cedars Model	Holdout Test	All	366 (54.6%)	0.78 (0.73-0.83)	0.81 (0.76-0.86)	0.19 (0.18-0.21)
		Cardiac ICU	101 (61.4%)	0.79 (0.69-0.87)	0.86 (0.77-0.93)	0.20 (0.17-0.22)
		Medical ICU	113 (43.4%)	0.74 (0.64-0.83)	0.70 (0.56-0.83)	0.21 (0.18-0.23)
		Surgical ICU	103 (66.0%)	0.75 (0.65-0.84)	0.86 (0.79-0.93)	0.19 (0.16-0.22)
	External Validation	All	2149 (51.8%)	0.58 (0.56-0.61)	0.60 (0.57-0.63)	0.25 (0.24-0.25)
		Cardiac ICU	159 (50.9%)	0.60 (0.52-0.69)	0.62 (0.51-0.73)	0.25 (0.22-0.27)
		Medical ICU	605 (42.6%)	0.55 (0.51-0.60)	0.47 (0.41-0.53)	0.26 (0.25-0.27)
		Surgical ICU	1093 (62.5%)	0.58 (0.55-0.62)	0.69 (0.65-0.73)	0.23 (0.23-0.24)
UCLA+ Cedars Model	Holdout Test	All	785 (52.7%)	0.82 (0.79-0.85)	0.84 (0.81-0.87)	0.17 (0.16-0.19)
		Cardiac ICU	145 (53.1%)	0.78 (0.70-0.85)	0.80 (0.71-0.88)	0.19 (0.17-0.22)
		Medical ICU	208 (36.5%)	0.78 (0.72-0.84)	0.67 (0.56-0.77)	0.19 (0.17-0.22)
		Surgical ICU	328 (66.5%)	0.85 (0.81-0.89)	0.92 (0.89-0.95)	0.15 (0.14-0.17)
	Holdout Test Stratified by UCLA	All	418 (52.9%)	0.85 (0.82-0.89)	0.87 (0.83-0.91)	0.16 (0.14-0.17)
		Cardiac ICU	33 (48.5%)	0.76 (0.58-0.92)	0.81 (0.63-0.94)	0.20 (0.15-0.25)
		Medical ICU	117 (35.0%)	0.83 (0.75-0.90)	0.70 (0.55-0.82)	0.18 (0.14-0.21)
		Surgical ICU	213 (68.1%)	0.88 (0.83-0.92)	0.94 (0.91-0.96)	0.14 (0.12-0.16)
	Holdout Test Stratified by Cedars	All	367 (52.6%)	0.78 (0.73-0.82)	0.80 (0.74-0.85)	0.19 (0.18-0.21)
		Cardiac ICU	112 (54.5%)	0.79 (0.70-0.87)	0.81 (0.70-0.89)	0.19 (0.16-0.22)
		Medical ICU	91 (38.5%)	0.72 (0.60-0.82)	0.65 (0.48-0.79)	0.21 (0.18-0.24)
		Surgical ICU	115 (63.5%)	0.80 (0.71-0.88)	0.88 (0.80-0.94)	0.18 (0.16-0.21)
All Datasets Model	Holdout Test	All	991 (38.7%)	0.85 (0.82-0.87)	0.78 (0.74-0.82)	0.15 (0.14-0.17)
		Cardiac ICU	147 (51.7%)	0.81 (0.74-0.88)	0.83 (0.75-0.90)	0.18 (0.15-0.22)
		Medical ICU	228 (40.4%)	0.71 (0.64-0.77)	0.67 (0.58-0.74)	0.22 (0.19-0.25)
		Surgical ICU	297 (62.0%)	0.79 (0.74-0.84)	0.85 (0.80-0.90)	0.19 (0.16-0.21)
	Holdout Test Stratified by UCLA	All	429 (50.8%)	0.77 (0.73-0.81)	0.79 (0.74-0.83)	0.20 (0.18-0.22)
		Cardiac ICU	38 (55.3%)	0.69 (0.52-0.85)	0.77 (0.60-0.91)	0.25 (0.17-0.33)
		Medical ICU	132 (40.2%)	0.75 (0.67-0.83)	0.72 (0.60-0.82)	0.20 (0.16-0.24)
		Surgical ICU	212 (61.3%)	0.78 (0.72-0.84)	0.85 (0.79-0.90)	0.20 (0.17-0.23)
	Holdout Test Stratified by Cedars	All	340 (48.8%)	0.79 (0.74-0.84)	0.78 (0.71-0.84)	0.19 (0.16-0.21)
		Cardiac ICU	109 (50.5%)	0.86 (0.78-0.93)	0.87 (0.77-0.94)	0.15 (0.12-0.19)
		Medical ICU	96 (40.6%)	0.63 (0.51-0.73)	0.57 (0.42-0.71)	0.24 (0.20-0.28)
		Surgical ICU	85 (63.5%)	0.83 (0.72-0.92)	0.86 (0.74-0.96)	0.16 (0.12-0.21)

Supplementary Table 3 Breakdown of Statistical Tests Used Based on Feature Characteristics

	Variable Type	Statistical Test Name	Effect Size Formula
Continuous	$X \sim \mathcal{N}^1$	t test	Hedges g
	$x \nsim \mathcal{N}^1$	Mann-Whitney U test	
Categorical	Binary	Fisher's Exact test	Cohen's h
	Multicategory	χ^2 test	Cramer's v

[1]$\mathcal{N}$ indicates the Normal or Gaussian distribution.

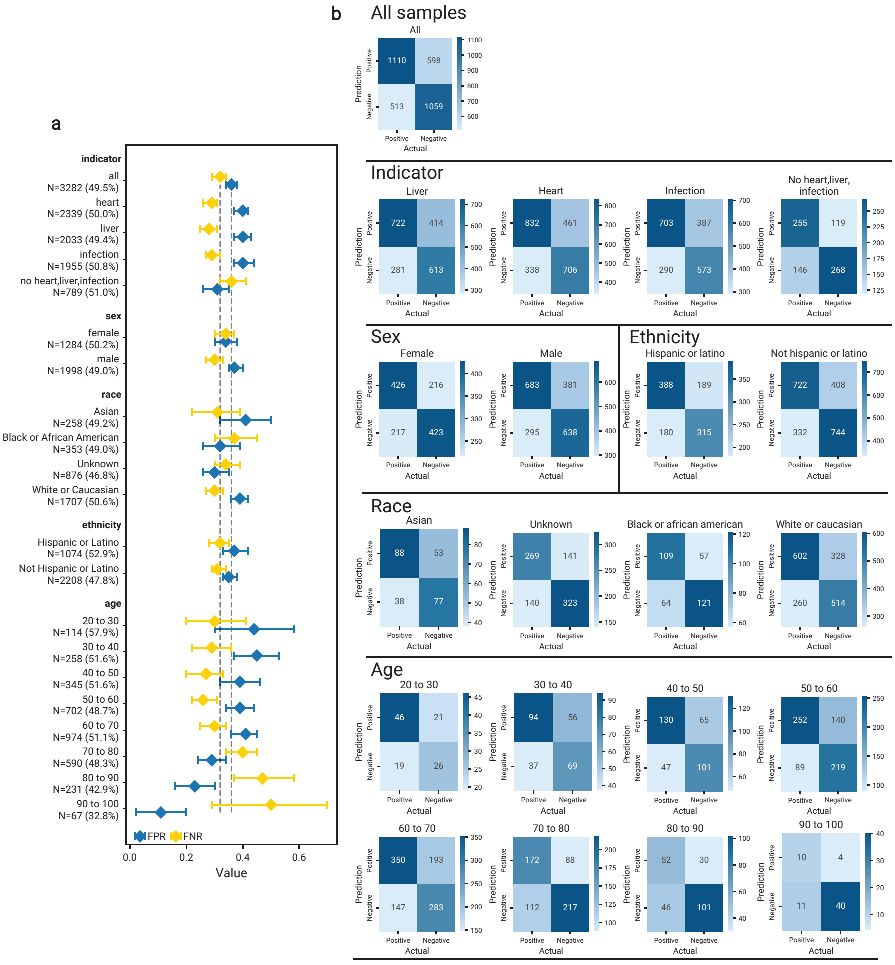

Supplementary Fig. 1 Bias analysis for the model trained and evaluated on a combination of the UCLA: CRRT, Cedars: CRRT, and UCLA: Control cohorts, using only features that existed across all three cohorts (defined in Section 2.2). a) False positive rate (blue) and false negative rates (yellow) are reported for a holdout test set ($N = 3,282$) including patients who were on CRRT for more than seven days. False positive rates and false negative rates are also reported when applying the model to subgroups of the test set. Subgroups were categorized by disease indicator, sex, race, ethnicity, and age. The reported statistics include point estimates as well as 95% confidence intervals obtained from 1,000 bootstrap iterations of the test dataset. b) Confusion matrices at a decision threshold of 0.5 when applying the model to same subgroups of the test set. Source data are provided as a Source Data file.

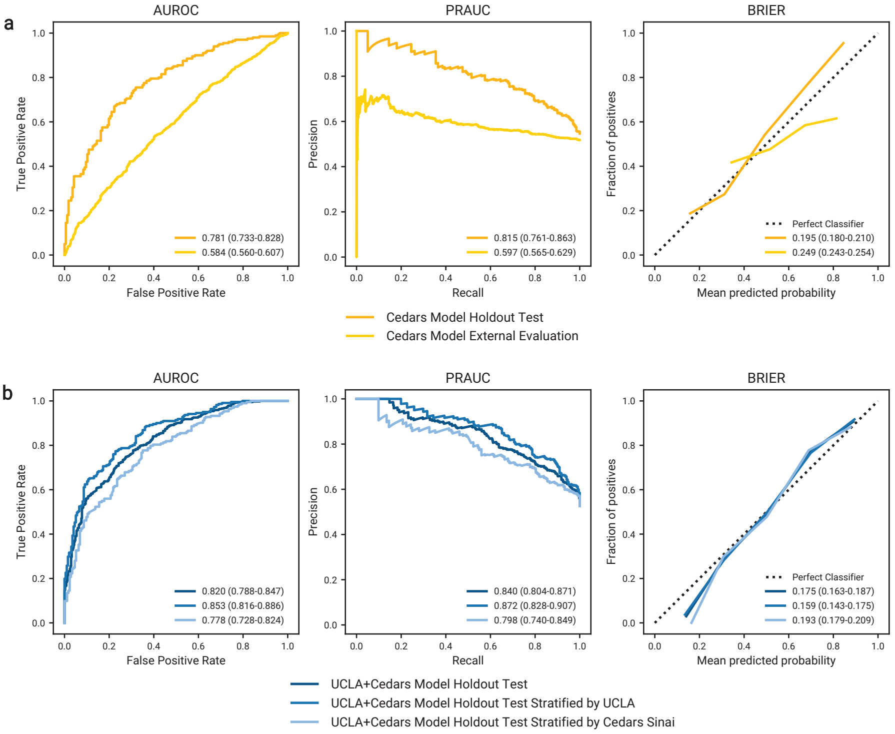

Supplementary Fig. 2 Model performance when predicting CRRT patient outcomes. The first column illustrates receiver operating characteristic curves for the prediction of CRRT outcome, with ROCAUC as the summarizing metrix. The second column illustrates precision curves for the prediction of CRRT outcome, with PRAUC as the summarizing metric. The third column illustrates calibration curves for the prediction of CRRT outcome, with the Brier score as the summarizing metrix. The reported statistics include point estimates as well as 95% confidence intervals obtained from 1,000 bootstrap iterations of the test dataset. a) The performance of a model trained on single-institution data from Cedars: CRRT ($N = 1,073$), evaluated on both an internal holdout test dataset ($N = 366$) shown in darker yellow, and an external dataset from UCLA: CRRT ($N = 2,149$) shown in lighter yellow. b) Performance of a model on the holdout test dataset ($N = 785$) after training on a combination of UCLA: CRRT and Cedars: CRRT cohorts ($N = 2,354$). The darkest blue curve illustrates the overall performance on the holdout test set, while the lighter and lightest blue curves illustrate the stratified results on the UCLA: CRRT ($N = 418$) and Cedars: CRRT ($N = 367$) constituents of the test dataset. Source data are provided as a Source Data file.

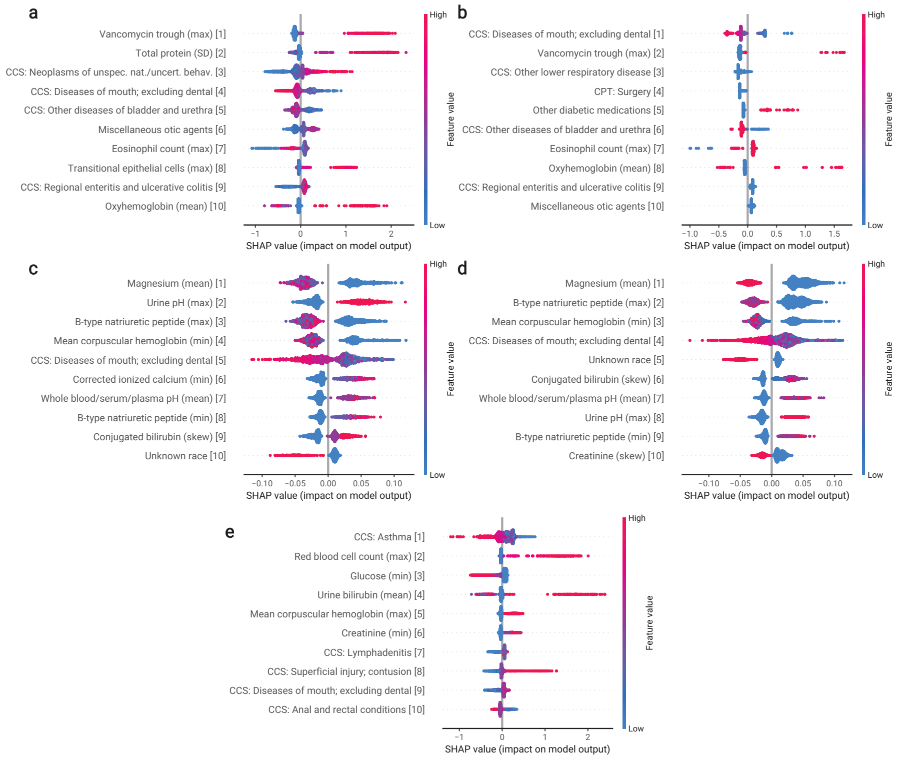

Supplementary Fig. 3 Ordered ranking of the ten most important features by average magnitude of SHAP values and direction of influence on output predictions. SHAP values were evaluated using holdout test sets that included patients who were on CRRT for more than seven days. Red indicates that a higher feature value has the corresponding impact, as indicated by the x-axis, on model output. Blue indicates the impact of lower feature values on model output. a) Model trained on data from UCLA: CRRT ($N = 1,268$), and evaluated on a holdout test set ($N = 1,746$). b) Model trained on data from UCLA: CRRT ($N = 1,268$), and evaluated on an external test set from Cedars: CRRT ($N = 2,867$). c) Model trained on data from Cedars: CRRT ($N = 1,073$), and evaluated on a holdout test set ($N = 1,316$). d) Model trained on data from Cedars: CRRT ($N = 1,073$), and evaluated on an external test set from UCLA: CRRT ($N = 3,698$). e) Model trained on a combination of data from UCLA: CRRT and Cedars: CRRT ($N = 2,354$), and evaluated on a holdout test set ($N = 3,092$).

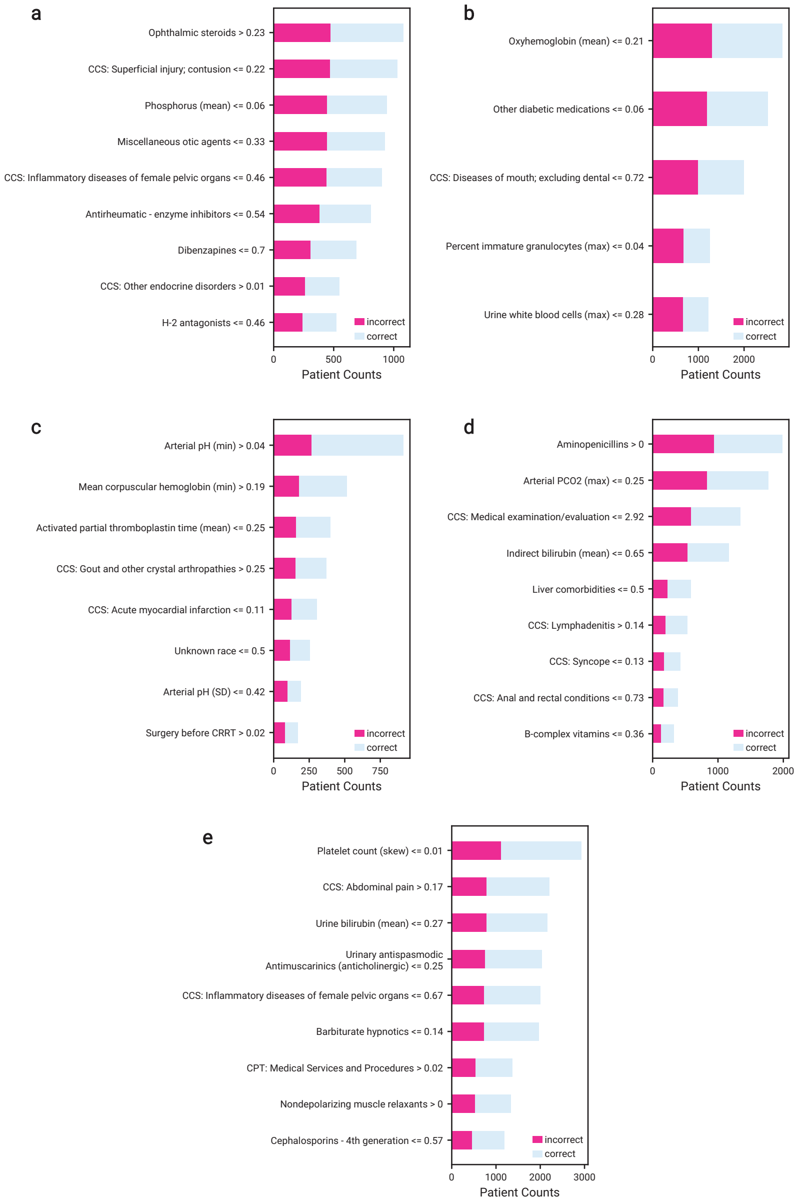

Supplementary Fig. 4 Top features that contributed to the majority of the errors. The threshold applied at each row operated on the resulting population after applying the respective threshold in the immediately above row. Pink indicates the number of incorrectly classified samples as a result of the decision threshold, while blue indicates the number of correctly classified samples. We performed error analysis on holdout test sets that included patients who were on CRRT for more than seven days. a) Model trained on data from UCLA: CRRT ($N = 1,268$), and evaluated on a holdout test set ($N = 1,746$). b) Model trained on data from UCLA: CRRT ($N = 1,268$), and evaluated on an external test set from Cedars: CRRT ($N = 2,867$). c) Model trained on data from Cedars: CRRT ($N = 1,073$), and evaluated on a holdout test set ($N = 1,316$). d) Model trained on data from Cedars: CRRT ($N = 1,073$), and evaluated on an external test set from UCLA: CRRT ($N = 3,698$). e) Model trained on a combination of data from UCLA: CRRT and Cedars: CRRT ($N = 2,354$), and evaluated on a holdout test set ($N = 3,092$). Source data are provided as a Source Data file.

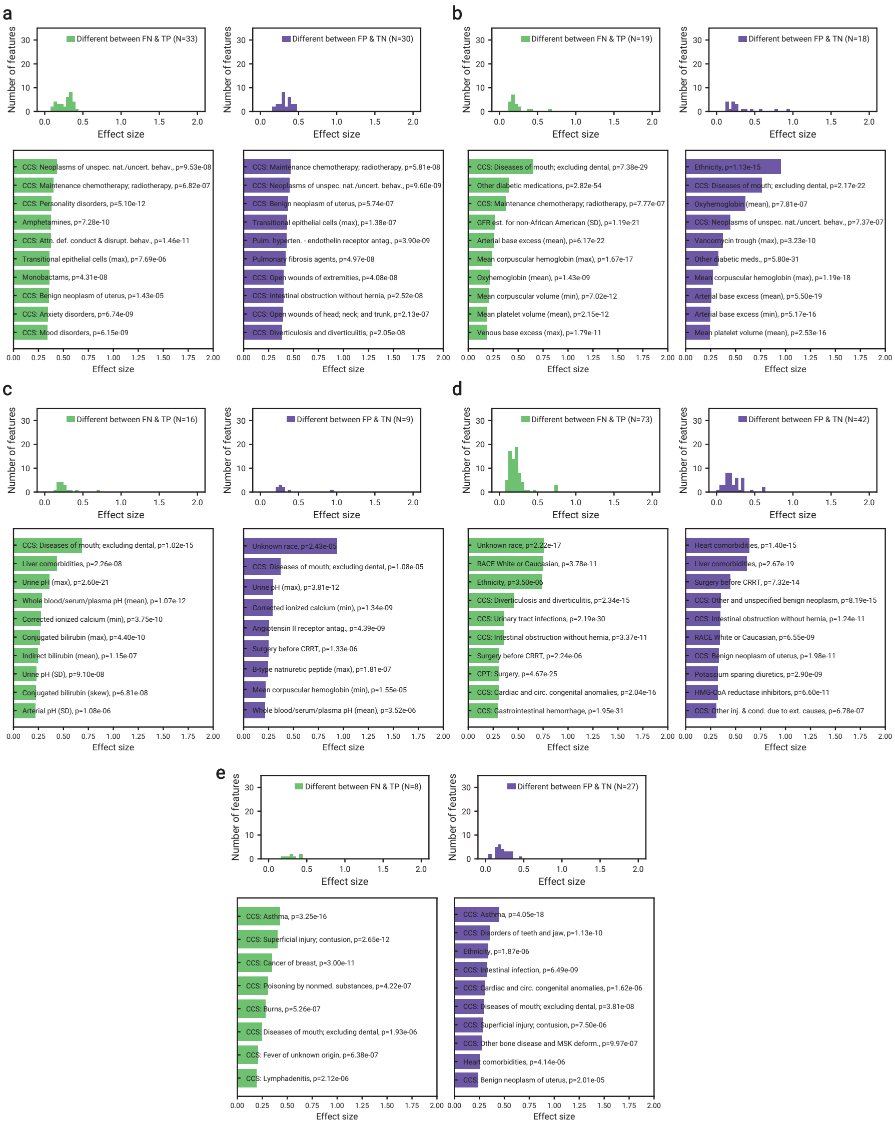

Supplementary Fig. 5 Summary of analysis of model randomness against feature noise. Effect sizes are shown for the features that were significantly different between false negative and true positive populations (green). Effect sizes are also shown for the features that were significantly different between false positive and true negative populations (purple). Features with the top ten effect sizes are shown. We performed analysis on holdout test sets that included patients who were on CRRT for more than seven days. Details of statistical test are described in 4.8. a) Model trained on data from UCLA: CRRT ($N = 1,268$), and evaluated on a holdout test set ($N = 1,746$). b) Model trained on data from UCLA: CRRT ($N = 1,268$), and evaluated on an external test set from Cedars: CRRT ($N = 2,867$). c) Model trained on data from Cedars: CRRT ($N = 1,073$), and evaluated on a holdout test set ($N = 1,316$). d) Model trained on data from Cedars: CRRT ($N = 1,073$), and evaluated on an external test set from UCLA: CRRT ($N = 3,698$). e) Model trained on a combination of data from UCLA: CRRT and Cedars: CRRT ($N = 2,354$), and evaluated on a holdout test set ($N = 3,092$). Source data are provided as a Source Data file.

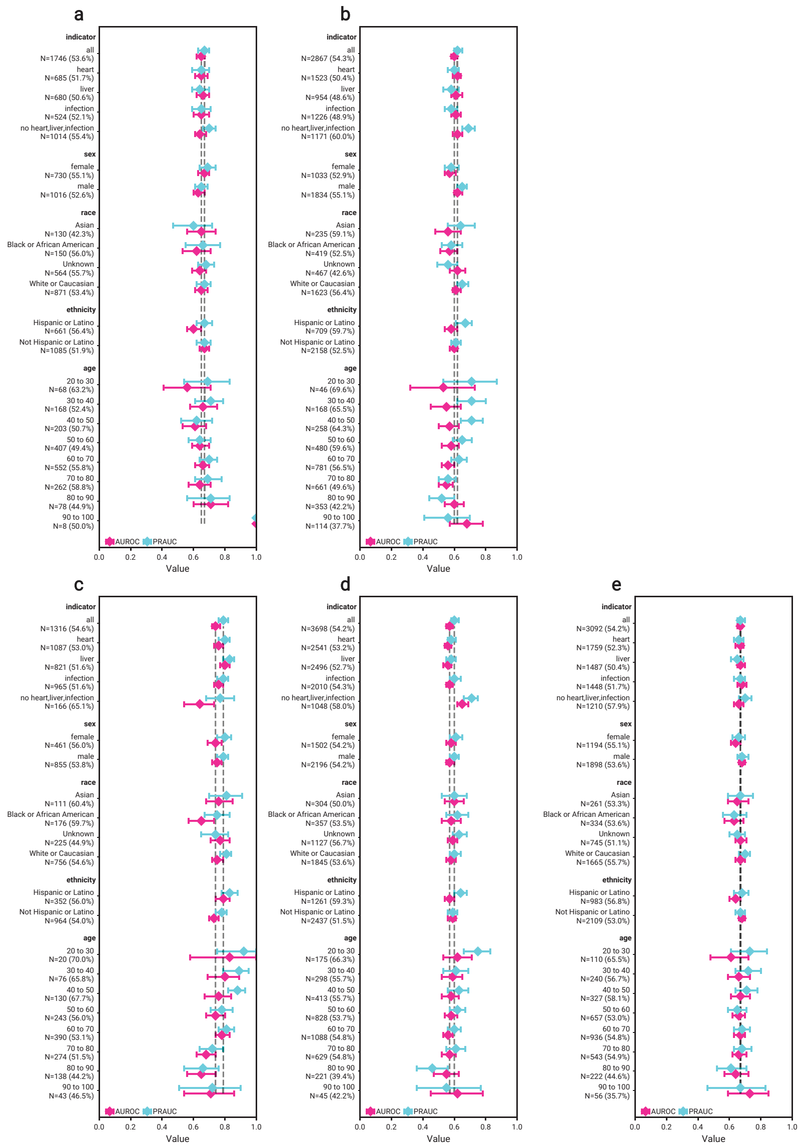

Supplementary Fig. 6 Performance measured by ROCAUC (pink) and PRAUC (blue) when applying models to subgroups of respective test sets. Subgroups were categorized by disease indicator, sex, race, ethnicity, and age. All test sets included patients who were on CRRT for more than seven days. Reported statistics include point estimates as well as as 95% confidence intervals obtained from 1,000 bootstrap iterations of the test dataset. a) Performance after training on data from UCLA: CRRT ($N = 1,268$), and evaluating on a holdout test set ($N = 1,746$). b) Performance after training on data from UCLA: CRRT ($N = 1,268$), and evaluating on an external test set from Cedars: CRRT ($N = 2,867$). c) Performance after training on data from Cedars: CRRT ($N = 1,073$), and evaluating on a holdout test set ($N = 1,316$). d) Performance after training on data from Cedars: CRRT ($N = 1,073$), and evaluating on an external test set from UCLA: CRRT ($N = 3,698$). e) Performance after training on a combination of data from UCLA: CRRT and Cedars: CRRT ($N = 2,354$), and evaluating on a holdout test set ($N = 3,092$). Source data are provided as a Source Data file.

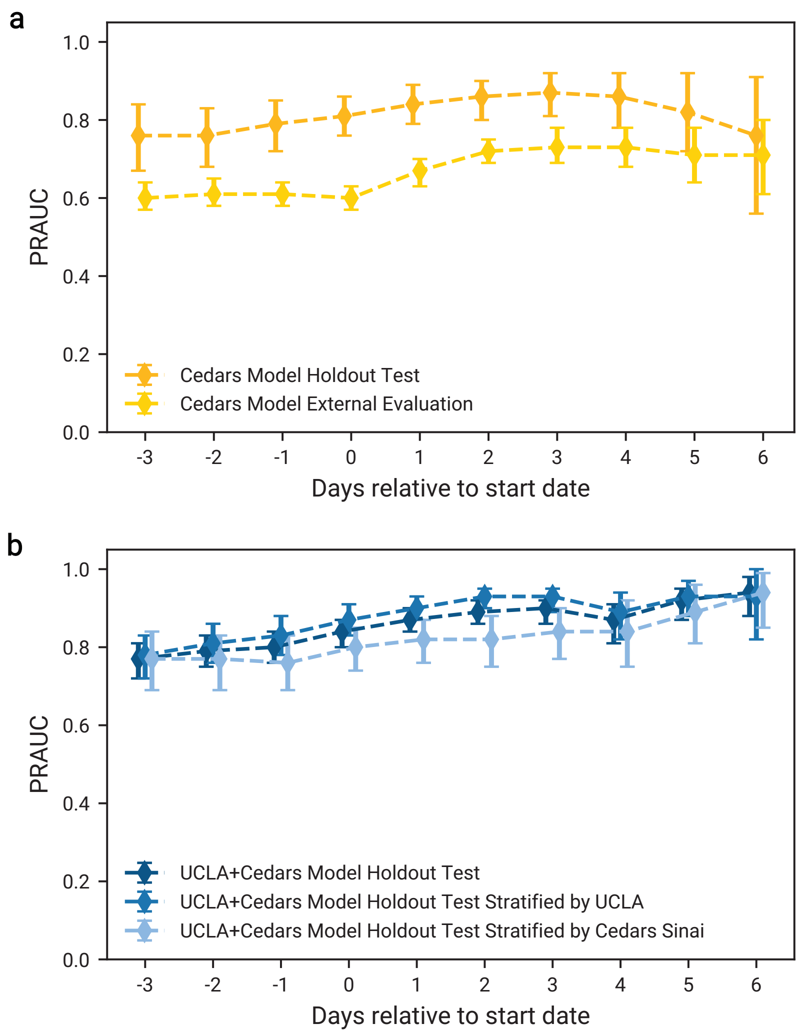

Supplementary Fig. 7 Model evaluation when using features from shifted windows between three days before and six days after the start date. All test sets were limited to patients who were on CRRT for within seven days. Reported statistics include PRAUC as well as as 95% confidence intervals obtained from 1,000 bootstrap iterations of the test dataset. a) Model trained on data from Cedars: CRRT ($N = 1,073$), evaluated on both an internal holdout test dataset ($N = 366$) shown in darker yellow, and an external dataset from Cedars: CRRT ($N = 2,149$) shown in lighter yellow. b) Model trained on a combination of data from UCLA: CRRT and Cedars: CRRT ($N = 2,354$). The darkest blue curve illustrates the overall performance on the holdout test set ($N = 785$), while the lighter and lightest blue curves illustrate the stratified results on the UCLA: CRRT ($N = 418$) and Cedars: CRRT ($N = 367$) constituents of the test dataset. Source data are provided as a Source Data file.

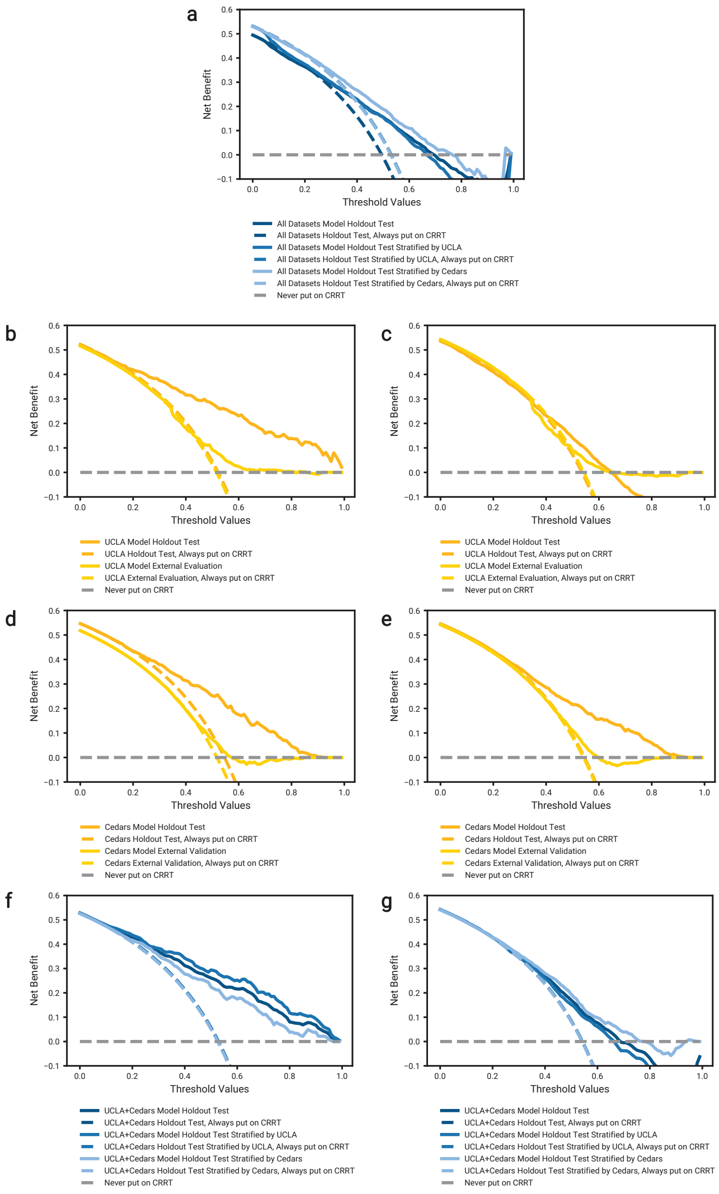

Supplementary Fig. 8 Decision curve analysis illustrating net benefit at different operating thresholds. a) Model trained on a combination of data from UCLA: CRRT, Cedars: CRRT, and UCLA: Control, and evaluated on a holdout test set including patients who were on CRRT for more than seven days ($N = 3,382$). b) Model trained on data from UCLA: CRRT ($N = 1,268$), and evaluated on a holdout test set ($N = 425$) and an external test set from Cedars: CRRT ($N = 1,788$). c) Same model as (b), but the test set included patients who were on CRRT for more than seven days ($N = 1,746$ and $N = 2,867$, respectively). d) Model trained on data from Cedars: CRRT ($N = 1,073$), and evaluated on a holdout test set ($N = 366$) and an external test set from UCLA: CRRT ($N = 2,149$). e) Same model as (c), but the test set included patients who were on CRRT for more than seven days ($N = 1,316$ and $N = 3,698$, respectively). f) Model trained on a combination of data from UCLA: CRRT and Cedars: CRRT ($N = 2,354$), and evaluated on a holdout test set ($N = 785$). g) Same model as (f), but the test set included patients who were on CRRT for more than seven days ($N = 3,092$). Source data are provided as a Source Data file.
