# Supplementary material for: Data-driven prediction of continuous renal replacement therapy survival
Source: Nat Commun. 2024 Jun 27;15:5440. doi: 10.1038/s41467-024-49763-3 (PMC11211317; doi:10.1038/s41467-024-49763-3)
Supplement: Supplementary file 3 — Reporting Summary [file 41467_2024_49763_MOESM3_ESM.pdf]

nature portfolio

Corresponding author(s): Dr. Alex A. T. Bui

Last updated by author(s): Jun 12, 2024

Reporting Summary

Nature Portfolio wishes to improve the reproducibility of the work that we publish. This form provides structure for consistency and transparency in reporting. For further information on Nature Portfolio policies, see our Editorial Policies and the Editorial Policy Checklist.

Statistics

For all statistical analyses, confirm that the following items are present in the figure legend, table legend, main text, or Methods section.

n/a	Confirmed	
☐	☒	The exact sample size (n) for each experimental group/condition, given as a discrete number and unit of measurement
☐	☒	A statement on whether measurements were taken from distinct samples or whether the same sample was measured repeatedly
☐	☒	The statistical test(s) used AND whether they are one- or two-sided<br>*Only common tests should be described solely by name; describe more complex techniques in the Methods section.*
☐	☒	A description of all covariates tested
☐	☒	A description of any assumptions or corrections, such as tests of normality and adjustment for multiple comparisons
☐	☒	A full description of the statistical parameters including central tendency (e.g. means) or other basic estimates (e.g. regression coefficient) AND variation (e.g. standard deviation) or associated estimates of uncertainty (e.g. confidence intervals)
☐	☒	For null hypothesis testing, the test statistic (e.g. F, t, r) with confidence intervals, effect sizes, degrees of freedom and P value noted<br>*Give P values as exact values whenever suitable.*
☒	☐	For Bayesian analysis, information on the choice of priors and Markov chain Monte Carlo settings
☒	☐	For hierarchical and complex designs, identification of the appropriate level for tests and full reporting of outcomes
☐	☒	Estimates of effect sizes (e.g. Cohen's d, Pearson's r), indicating how they were calculated

Our web collection on statistics for biologists contains articles on many of the points above.

Software and code

Policy information about availability of computer code

Data collection	Data collection was completed using the Oracle database housing Electronic Health Records (HER) data as the source. The Oracle version used was Oracle Database 19c Enterprise Edition Release 19.0.0.0.0 – Production Version 19.17.0.0.0. The data was obtained and queried using SQL Developer Version 21.2.1.204 and Oracle SQL*Plus Release 18.0.0.0.0 - Production Version 18.3.0.0.0.
Data analysis	We implemented our procedure in Python 3.9.16. We primarily use the NumPy 1.23.0 and pandas 1.5.3 packages for loading and manipulating our datasets. Models are implemented via lightgbm 3.3.5 for an implementation of LGBM, xgboost 1.7.4 for an implementation of XGB, and Scikit-learn 1.2.2 for all other models. We use the Optuna 3.2.0 framework for model tuning and the algorithm for sampling from the hyperparameter grid. Statistical tests are computed using the SciPy 1.10.1 and Scikit-learn 1.2.2 packages. We use Shapley additive explanations (SHAP) 0.42.0 for generating feature importance and explaining model decision-making on particular samples. Lastly, we use the Mealy 0.2.5 package for visualizing model errors. The corresponding code can be found at https://github.com/uclamii/CRRT.

For manuscripts utilizing custom algorithms or software that are central to the research but not yet described in published literature, software must be made available to editors and reviewers. We strongly encourage code deposition in a community repository (e.g. GitHub). See the Nature Portfolio guidelines for submitting code & software for further information.

Data

Policy information about availability of data

All manuscripts must include a data availability statement. This statement should provide the following information, where applicable:

- Accession codes, unique identifiers, or web links for publicly available datasets
- A description of any restrictions on data availability
- For clinical datasets or third party data, please ensure that the statement adheres to our policy

We obtained approval from the UCLA Institutional Review Board (Protocol Number 19-00093) to collect de-identified data from UCLA and Cedars Sinai Medical Center. UCLA IRB waived the need for consent on the use of retrospective data collected from routine clinical processes. As: (1) patients did not provide consent to participate in this study; and (2) the datasets used in this study are considered property of two health systems (UCLA, Cedars-Sinai), the data from this study can only be released to any third party by permission from our institutional Health Data Oversight Committee (HDOC). The policies and procedures that UCLA HDOC uses are based on revised guidelines put forth by the University of California (UC) on May 1, 2024. Requests for data releases can be made to Sandy Binder (SLBinder@mednet.ucla.edu), who is the UCLA HDOC Administrator. Requests will be evaluated against the five principles: (1) attention to the University's unique responsibility and mission; (2) sharing data outside UC for public benefit, (3) justice, (4) transparency and patient engagement, and (5) responsible stewardship. UCLA believes in a collaborative approach to enhancing reproducible science and encourages data sharing following its policies and procedures. HDOC reviews requests to ensure no apparent sale or barter of health data in exchange for goods, services, or other benefits; limit redisclosure or reuse of health data with additional third parties without restriction; health data requests do not extend beyond the minimum necessary required to answer specific scientific hypotheses and that the request does not involve large volumes of data dealing with sensitive populations; sharing of potentially identifiable biometric data; conflicts of interest; and unusual or unique terms in the data use agreement that might pose risk to UC or patient population. All data releases require a data use agreement. HDOC meets biweekly and requests can usually be reviewed within 2-4 weeks. Once HDOC approves a data release, UCLA's Technology Development Group will work to complete a data use agreement and provision the data. Source data are provided with this paper.

Research involving human participants, their data, or biological material

Policy information about studies with human participants or human data. See also policy information about sex, gender (identity/presentation), and sexual orientation and race, ethnicity and racism.

Reporting on sex and gender	Sex was obtained from the Oracle database housing EHR data. There are 5 possible values for Sex: Male, Female, Unknown, Other, and X. These values are reported by the patient. Gender identity, which is also reported by the patient, is an additional available variable in the EHR data, but it was not delivered as part of the analysis. Sex was used as input to our machine learning models and for a fairness and bias analysis.
Reporting on race, ethnicity, or other socially relevant groupings	Race and ethnicity were obtained from the Oracle database housing EHR data. Each race and ethnicity is reported by the patient. However, multiple races were programmatically merged using custom logic. Race and ethnicity were used as inputs to our machine learning models and as variables for our fairness/bias analysis.
Population characteristics	Population characteristics used for analyses include patient age, sex, race and ethnicity, as well as allergies, full list of past and current diagnoses, medications prescribed and taken, labs ordered and completed, and procedures completed. Age of UCLA and Cedars patients had a median of 60 and 66 respectively. 59.6% and 63.7% of patients from UCLA and Cedars respectively were male. 66% and 75.3% of patients from UCLA and Cedars respectively were non-Hispanic or Latino.
Recruitment	Participants were not prospectively recruited for this research.
Ethics oversight	Office of the Human Research Protection Program. We obtained approval from the UCLA Institutional Review Board (Protocol Number 19-00093) to collect de-identified data from UCLA and Cedars Sinai Medical Center. UCLA IRB waived the need for consent on the use of retrospective data collected from routine clinical processes.

Note that full information on the approval of the study protocol must also be provided in the manuscript.

Field-specific reporting

Please select the one below that is the best fit for your research. If you are not sure, read the appropriate sections before making your selection.

☒ Life sciences ☐ Behavioural & social sciences ☐ Ecological, evolutionary & environmental sciences

For a reference copy of the document with all sections, see nature.com/documents/nr-reporting-summary-flat.pdf

Life sciences study design

All studies must disclose on these points even when the disclosure is negative.

Sample size	There were no predetermined sample sizes. Data was collected from UCLA and Cedars Sinai Medical Center between 2014 and 2021, resulting in 12,149 total samples.
Data exclusions	Pediatric cases of continuous renal replacement therapy were excluded (less than 21 years of age).
Replication	Our experimental findings involving machine-learning models were rigorously and successfully evaluated using proper machine-learning practices, including hyper-parameter tuning with a validation dataset, evaluating on hold-out test sets, and demonstration of performance

across multiple institutions (external testing).

Randomization	Randomization was not relevant to the study. From a machine-learning perspective, train/validation/testing splits were randomly generated.
Blinding	Blinding was not relevant to the study.

Reporting for specific materials, systems and methods

We require information from authors about some types of materials, experimental systems and methods used in many studies. Here, indicate whether each material, system or method listed is relevant to your study. If you are not sure if a list item applies to your research, read the appropriate section before selecting a response.

Materials & experimental systems

n/a	Involved in the study
☒	☐ Antibodies
☒	☐ Eukaryotic cell lines
☒	☐ Palaeontology and archaeology
☒	☐ Animals and other organisms
☐	☒ Clinical data
☒	☐ Dual use research of concern
☒	☐ Plants

Methods

n/a	Involved in the study
☒	☐ ChIP-seq
☒	☐ Flow cytometry
☒	☐ MRI-based neuroimaging

Clinical data

Policy information about clinical studies

All manuscripts should comply with the ICMJEguidelines for publication of clinical research and a completedCONSORT checklist must be included with all submissions.

Clinical trial registration	Research involved clinical data but was not part of a trial.
Study protocol	Research involved clinical data but was not part of a trial.
Data collection	Research involved clinical data but was not part of a trial. Data was collected from UCLA and Cedars Sinai Medical Center between 2014 and 2021.
Outcomes	Research involved clinical data but was not part of a trial. Outcomes included binary targets for renal function recovery, transition to hemodialysis, transition to comfort care, or expiration.

Plants

Seed stocks	*Report on the source of all seed stocks or other plant material used. If applicable, state the seed stock centre and catalogue number. If plant specimens were collected from the field, describe the collection location, date and sampling procedures.*
Novel plant genotypes	*Describe the methods by which all novel plant genotypes were produced. This includes those generated by transgenic approaches, gene editing, chemical/radiation-based mutagenesis and hybridization. For transgenic lines, describe the transformation method, the number of independent lines analyzed and the generation upon which experiments were performed. For gene-edited lines, describe the editor used, the endogenous sequence targeted for editing, the targeting guide RNA sequence (if applicable) and how the editor was applied.*
Authentication	*Describe any authentication procedures for each seed stock used or novel genotype generated. Describe any experiments used to assess the effect of a mutation and, where applicable, how potential secondary effects (e.g. second site T-DNA insertions, mosiacism, off-target gene editing) were examined.*
